# Two Decades from the Introduction of Microdissection Testicular Sperm Extraction: How This Surgical Technique Has Improved the Management of NOA

**DOI:** 10.3390/jcm10071374

**Published:** 2021-03-29

**Authors:** Nahid Punjani, Caroline Kang, Peter N. Schlegel

**Affiliations:** Department of Urology, Weill Cornell Medical College, New York, NY 10065, USA; nap4001@med.cornell.edu (N.P.); cak4005@med.cornell.edu (C.K.)

**Keywords:** microdissection testicular sperm extraction, non-obstructive azoospermia, management

## Abstract

The treatment of men with non-obstructive azoospermia (NOA) has improved greatly over the past two decades. This is in part due to the discovery of in vitro fertilization (IVF) and intracytoplasmic sperm injection (ICSI), but also significantly due to improvements in surgical sperm retrieval methods, namely the development of microdissection testicular sperm extraction (mTESE). This procedure has revolutionized the field by allowing for identification of favorable seminiferous tubules while simultaneously limiting the amount of testicular tissue removed. Improving sperm retrieval rates is imperative in this cohort of infertile men as there are a limited number of factors that are predictive of successful sperm retrieval. Currently, sperm retrieval in NOA men remains dependent on surgeon experience, preoperative patient optimization and teamwork with laboratory personnel. In this review, we discuss the evolution of surgical sperm retrieval methods, review predictors of sperm retrieval success, compare and contrast the data of conventional versus mTESE, share tips for optimizing sperm retrieval outcomes, and discuss the future of sperm retrieval in men with NOA.

## 1. Introduction

Infertility affects up to 15% of couples attempting to conceive globally, with a male factor implicated in up to 50% of cases [1]. While the precise etiology remains unclear in many of these cases, azoospermia, or the lack of sperm in the ejaculate, occurs in 1% of all males and 10–15% of infertile males and is often considered the most severe phenotype of male infertility classified as either obstructive azoospermia (30–40% of azoospermia cases) or non-obstructive azoospermia (NOA) (60–70% of azoospermia cases) [2,3,4,5,6]. NOA remains a particularly challenging condition to treat as the majority of cases are idiopathic, with only a subset attributable to an identifiable genetic (i.e., Klinefelter Syndrome, Y-chromosome microdeletion or mutations in individual genes) or acquired (i.e., chemotherapy, radiation, cryptorchidism/prior orchiopexy or malignancy) condition [7,8]. NOA men have spermatogenic failure with a range of histopathologic changes that include hypospermatogenesis, maturation arrest, and Sertoli cell only syndrome [7]. Currently, NOA men require surgical retrieval of sperm with assisted reproductive technology to father children. This review highlights milestones in the evolution of surgical sperm retrieval methods, summarizes predictors of sperm retrieval success, evaluates the data of conventional microdissection testicular sperm extraction (cTESE) versus microdissection testicular sperm extraction (mTESE), discusses tips for optimizing sperm retrieval, and comments on the future of sperm retrieval in men with NOA.

## 2. History of Surgical Sperm Retrieval

**Figure 1:**
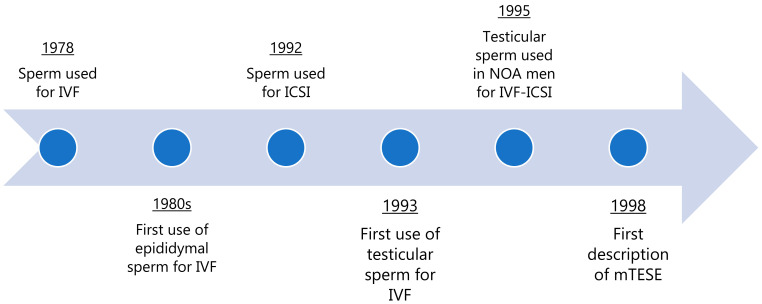
Timeline of the history of surgical sperm retrieval prior to the discovery of mTESE. IVF: in vitro fertilization; ICSI: intracytoplasmic sperm injection; NOA: non-obstructive azoospermia; mTESE: microdissection testicular sperm extraction.

While NOA men currently rely on surgical sperm retrieval with assisted reproductive technology to father biological children, these men were historically relegated to using adoption or use of donor sperm to have a family [9]. The first successful in vitro fertilization (IVF), the process by which a sperm and oocyte are fertilized outside of the body and then later implanted, was performed in 1978 utilizing ejaculated sperm from a fertile man, and resulted in the birth of Louise Brown (Figure 1) [10]. Sperm was surgically retrieved for the use of IVF for the first time in the 1980s, utilizing motile sperm from the epididymis of man with obstructive azoospermia [11]. Intracytoplasmic sperm injection (ICSI), a process where only a single sperm is injected into an oocyte using a micropipette, was then introduced in 1992, thereby potentially providing an opportunity for men with severe spermatogenic dysfunction (i.e., men with NOA) to father children [12]. The first description of testicular sperm for use in assisted reproduction occurred in 1993 [13]. Successful fertilization, embryo development, implantation and pregnancy was considered to be an unanticipated result by some reproductive experts given the expected need for additional maturation by sperm that was known to occur during epididymal transit [14].

It was not until 1995 that testicular sperm extraction (TESE), an open surgical procedure to directly extract testicular tissue, was performed on a man with NOA and testicular sperm utilized for successful IVF-ICSI [15]. This success was revolutionary for the treatment of men with NOA, but certain challenges remained given the sporadic, almost anecdotal, success of initial efforts to treat men with NOA who were previously considered to be sterile [16]. Based on observations that limited sperm was retrieved during simple biopsy, it was originally thought that multiple testis biopsies would increase the chance of retrieval as sperm production was believed to occur in isolated areas in the testis of men with NOA and spermatogenic failure [14]. In reality, multiple open biopsies resulted in removal of large quantities of testicular tissue and created a new risk, namely of harm to the blood supply of the testis from multiple incisions on the tunica albuginea. These incisions threatened to divide the vessels under the surface of the tunica vaginalis, with a potential risk of testis devascularization [17]. Percutaneous needle aspiration of the testis provided a minimally invasive alternative to sperm retrieval in NOA, but the lower sperm yield often did not provide enough sperm to inject all oocytes during an attempted ICSI attempt [18]. Given the risk of vascular injury, an approach to widely opening the testis and identifying individual tubules with the aid of an operating microscope was initiated. With this additional magnification, differences in seminiferous tubules could be visualized and appreciated, in particular differences shown to reflect potential focal sites of sperm production in an otherwise highly dysfunctional testis. Additional considerations of the intratesticular blood supply that runs parallel to seminiferous tubules within the testis allowed the development of microdissection testicular sperm extraction (mTESE) by Schlegel et al. in 1998 [19]. Additional studies have documented enhanced sperm retrieval rates with this technique, as well as the additional safety of mTESE, by eliminating the need for multiple biopsies or incisions of the tunica albuginea, reducing the impact to the testicular blood supply [20,21].

## 3. Surgical Testicular Sperm Extraction

**Figure 2:**
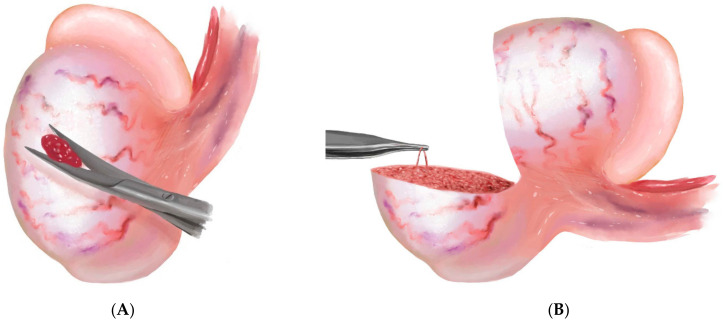
Graphical representation of a (**A**) conventional testicular sperm extraction with or without an operating microscope and (**B**) microdissection testicular sperm extraction using an operating microscope.

TESE is the surgical removal of tissue from the testicle in order to retrieve sperm, and can be completed with or without a standard operating microscope. cTESE may be completed with local anesthetic or sedation, but is commonly completed under general anesthesia. A skin incision may be made in the scrotal midline or through a unilaterally transverse or longitudinal incision over the selected hemiscrotum. Dissection is carried through subcutaneous dartos tissue down towards the tunica vaginalis which is then opened to reveal the testis. If delivered, the testis should be examined to identify and avoid areas of prominent vascularity, commonly seen in the midline and lower poles of the testis. An ultra-sharp or ophthalmic blade is used to sharply enter the tunica albuginea. Gentle pressure is applied around the tunical incision to extrude a sample of seminiferous tubules (Figure 2A). The tubules are sharply excised with scissors and processed in sperm-appropriate media by mincing the tissue with scissors and passage through a 24-gauge angiocatheter. The specimen is then examined by a trained andrologist, under light or phase-contrast microscopy at 20× magnification, for the presence of sperm.

mTESE is now considered the gold standard procedure for sperm retrieval in men with NOA [22]. Under general anesthesia, a dissection identical to cTESE is performed, and the testis is delivered through an opening in the parietal tunica vaginalis. After delivery of the testicle, adhesions on the visceral tunica vaginalis should be released to ensure optimal visualization and location of vasculature. The tunica vaginalis is then sharply incised with an ultra-sharp or ophthalmic blade, but bi-valved equatorially (Figure 2B). The tunical edges are secured with clamps to prevent avulsion of the tunica from the underlying tubules. The seminiferous tubules are then examined carefully and systematically prior to manipulation. Bipolar cautery should be used to limit postoperative bleeding as well as tissue damage. The tissue should be manipulated with care to avoid disruption of individual seminiferous tubules or the vessels which run parallel to the tubules in a radial pattern from the center to the periphery of the testicular parenchyma. Once an optimal tubule is identified, the tubule should be taken in its entirety, whenever possible. Optimal tubules (i.e., those likely to contain sperm) are generally larger and more opaque [20]. Once an adequate number of tubules are selected, processing should occur as previously described using sharp scissors and aspiration through a 24-gauge angiocatheter. This mechanical disruption of testicular tissue can allow detection of rare sperm within the tissue that may not be identifiable in a sample that has not been similarly processed. Note that sperm, when present, are inside the seminiferous tubules, so the tubules must be broken open to release sperm into the tissue suspension. As previously described, a limited part of this dispersed tissue specimen can then be examined by an experienced andrologist using 20× phase contrast microscopy on a simple slide with cover slip.

## 4. Predictors of Sperm Retrieval in mTESE

Although many studies have been conducted examining sperm retrieval rates in men with NOA undergoing mTESE, limited definitive predictors of sperm retrieval exist.

### 4.1. Histopathology

Histopathology is one of the strongest predictors of sperm retrieval as it provides a direct snapshot of the testicular architecture [23]. However, performance of a testicular biopsy solely for diagnostic purposes is not routinely recommended because of its invasiveness. In addition, a diagnostic biopsy samples only a small section of the testicular tissue, so its predictive value is limited. Diagnostic biopsy is suggested at the time of retrieval attempt to document the condition treated and rule out pathologic processes such as intratubular germ cell neoplasia. Additionally, testis biopsy may also be performed to differentiate maturation arrest from normal production in men with normal volume azoospermia, normal serum follicle stimulating hormone (FSH) concentrations, palpable vas deferens, and normal testicular volume [24].

Histopathologic subtype has been correlated to sperm retrieval rate, and those with Sertoli cell only syndrome histopathology have lower sperm retrieval rates compared to those with maturation arrest or hypospermatogenesis patterns [25]. As expected, the presence of mature spermatozoa is a strong predictor of sperm retrieval [26]. Unfortunately, despite the utility of testicular histopathology, it often is not available prior to sperm retrieval procedures, and is of limited value when no spermatozoa are seen [27]. For example, men with a diagnostic biopsy showing Sertoli cell only syndrome are expected to have sperm production elsewhere in the testis in at least 37% of cases. At our institution it is standard that pathology reports include all histologic patterns present in a testis biopsy, as even small foci of spermatogenesis are correlated with successful sperm retrieval. Pathology reports that state only the predominant or most severe histopathology are highly unlikely to reflect the likelihood of sperm retrieval in men with NOA.

### 4.2. Testis Size

In a meta-analysis by Corona et al., men with testis size >12 cc had higher rates of sperm retrieval, however, sperm retrieval was still possible in small volume testes (<8 cc) [28]. Our observations are the opposite; that men with larger testes are more likely to have obstructive azoospermia, as suggested by Schoor et al., and that men with NOA have similar sperm retrieval chances, regardless of testis volume [27]. With an effective mTESE search for rare foci of sperm, the testis volume or FSH level (which reflects overall testicular function) cannot predict the region of best function/sperm production inside the testis. Other meta-analyses have demonstrated limited predictive value of testicular volume even when testis biopsy histopathologic patterns were also used in the analysis [29]. Overall, these data suggest that testis size should not be considered a factor to exclude a patient from an attempt at sperm retrieval. In fact, in our experience, sperm can be routinely retrieved even in testes less than 2 cc in volume [30].

### 4.3. Serum Follicle Stimulating Hormone Levels

Serum FSH concentrations have been suggested, in some isolated reports, to predict sperm retrieval in conventional TESE [31]. Other studies have reported FSH levels to inversely correlate with the number of germ cells present and stages of spermatogenesis [32]. However, while high serum FSH levels may provide a more global representation of the level of spermatogenic dysfunction within a testis, there still may be small foci of spermatogenesis that can be identified and retrieved during mTESE [33]. Therefore, we do not recommend using baseline serum FSH concentrations as a preoperative predictor of sperm retrieval in NOA men.

### 4.4. Age

Male age has been correlated with deterioration in ejaculated semen parameters and increased serum FSH concentrations [34]. It has been proposed that FSH increases secondary to decreased androgen production associated with aging [35]. However, since FSH is not predictive of sperm retrieval, as discussed above, age also should not be a predictive factor [28]. Age has been suggested to be a predictive factor in certain patient subsets, such as those men with Klinefelter Syndrome, but at our center, sperm retrieval rates for Klinefelter Syndrome patients in the oldest age group (>35 years) are still 50%. These observations do not provide compelling evidence for early sperm retrieval [36]. It has been our uncontrolled observation that in many men with severe oligozoospermia followed longitudinally using repeated semen analyses that it is rare for these men to progress to azoospermia during our observation period. Finally, while advanced paternal age has reported negative impact on outcomes in offspring (increased genetic risks), our experience has demonstrated no upper age limit (even men in their 80s) for successful sperm retrieval [37].

### 4.5. Genetics

The genetic makeup of a man with NOA may provide insight into his chances of successful sperm retrieval and can aid greatly in preoperative counseling. As per guidelines from the American Society of Reproductive Medicine and American Urologic Association, men with NOA or severe oligozoospermia (<5 million sperm/mL) should undergo karyotype analysis and screening for Y chromosome microdeletions [22,24]. It is well-known that the presence and location of a Y chromosome microdeletion in a man with NOA is helpful in predicting the chance of sperm retrieval [38]. Men with complete AZFa and AFZb deletions have sperm retrieval rates of zero, whereas men with AZFc deletions have reported sperm retrieval rates of up to 70% [38,39]. Furthermore, detection of Klinefelter Syndrome (47,XXY) provides favorable prognostic information, as these men tend to have similar or better rates of sperm retrieval as compared to other NOA men (ranging from 65–70% retrieval rates at our center over time) [9,37]. Moving forward, improved diagnostics such as whole exome sequencing may identify specific genetic abnormalities that may provide further prognostic information related to sperm retrieval success [40,41].

### 4.6. History of Cryptorchidism/Orchiopexy

Most men with a history of cryptorchidism have sperm in their ejaculate and nearly normal fertility (unilateral cryptorchidism) or mildly impaired fertility (bilateral cryptorchidism). Therefore, men who are azoospermic with a history of cryptorchidism are relatively unique. Our experience has demonstrated that men with azoospermia and a history of cryptorchidism/orchiopexy have unique anatomic features. These men often have a testis that is in a different anatomic configuration with the epididymis anterior, and the caput epididymis that may be inferior within the scrotum. Taken together with the lack of tunica vaginalis that is typically present after orchiopexy, exploration of the scrotum in these men can be difficult. Care must be taken to identify each anatomic structure, with special care taken to identify the testicular blood supply. The surface of the testis in these patients often has very prominent vessels that course in a longitudinal fashion, almost suggesting a pattern of neovascularity. In some cases, the primary testicular blood supply does not enter in the standard location just medial to the caput epididymis.

Since most pexed testes have good sperm production, the azoospermic man with cryptorchidism/orchiopexy has a very different spermatogenic picture. Given the intraoperative observations we have made, it is possible that some of these men are azoospermic because of an alteration in testicular blood supply that occurred during, or as a consequence of, surgical orchiopexy.

Only limited reports of sperm retrieval success after orchiopexy for cryptorchidism have been published to-date, as of 2020 [42,43,44,45,46]. In these studies, combinations of testicular volume, unilateral and bilateral cryptorchidism have been debated as potential factors affecting the chance of sperm retrieval. Overall, a history of cryptorchidism has been suggested to be a favorable factor for sperm retrieval, with retrieval rates of 55–74% reported in these studies. We have typically found that most of the testis will be replaced with sclerotic tubules, with small distinct foci of sperm production, often in areas with Leydig cell hyperplasia (notable by the yellow color around the enlarged seminiferous tubules in these sites.)

### 4.7. Other Factors

Various studies have attempted to explore other factors including inhibin B levels, various gene products or transcripts in the ejaculate as well as anti-Müllerian hormone levels as predictors of successful sperm retrieval, but none have shown adequate association to warrant clinical application as predictors of sperm retrieval [37,47,48].

## 5. Outcomes of cTESE vs. mTESE

Numerous studies have endeavored to compare cTESE to mTESE. One of the first reviews summarized seven studies between 1999 and 2013 and reported sperm retrieval rates of 16.7–45.0% in the cTESE group and 42.9–63.0% in the mTESE group [49]. Shortly after, a meta-analysis of 15 studies with almost 2000 patients demonstrated that mTESE had a 1.5× greater likelihood of successful sperm retrieval compared to cTESE [47]. The strength of this meta-analysis was that it included only comparative studies, i.e., publications where the same selection of patients and same surgical/laboratory expertise was applied to compare patient outcomes in these settings. These data remain the most robust comparisons of testicular fine needle aspiration (TESA) with cTESE, showing a two-fold improvement in sperm retrieval rates with cTESE vs. TESA, as well as a higher rate of sperm retrieval with mTESE vs. cTESE (1.5× higher).

The review by Corona et al. published in 2019, included over 21,000 patients [28]. Since the authors included data from different patient cohorts with varying underlying etiologies for NOA, it is not possible to rely on the results tabulated as being a valid comparison of different sperm retrieval techniques. They did note a randomized trial which reported a retrieval rate of 42% (29/69 testicles) in the cTESE group versus 52% (36/69 testicles) in the mTESE group who had sperm retrieved at the time of surgery [48]. The authors suggest that this difference was due to an ability to view larger tubules, obtain tubules from more vascularized areas, and the ability to map the testicle during mTESE. The findings of this one prospective RCT (randomized control trial) should be considered strongly as the randomized study design reduces confounding and affords the greatest exchangeability between study groups. The quality of a meta-analysis is only as good as the studies for which it summarizes, and in this case consisted almost entirely of observational data with many possible sources of bias.

## 6. Optimizing Success

Since NOA men may have focal areas of sperm production within their testis, preoperative optimization is a key strategy for successful sperm retrieval during TESE. As spermatogenesis takes approximately 74 days, it is important to ensure that patients do not have surgical intervention to the testicle including biopsy for at least 6 months prior to their procedure [9]. Men with varicocele warrant consideration of possible varicocele repair, and may be considered in certain selected patients, especially couples with a younger female partner and lower FSH (less risk of Sertoli cell only syndrome) as well as those with previously documented sperm in the ejaculate or those with ample time to benefit from a return of sperm in their ejaculate. The caveat to varicocele repair is that it may take >6 months for return of sperm to the ejaculate, and only a small subset (<10%) may have adequate numbers of sperm to negate the need for surgical retrieval [9]. Again, of concern in these studies is the lack of a control group to compare to the patients who had varicocele repair for NOA. Some men with previously documented azoospermia will have rare sperm detected in a repeat semen analysis, especially if the concentrated pellet is more carefully examined (“extended sperm search”) [50].

Hormonally, men with NOA typically have elevated gonadotropins (i.e., FSH), low serum testosterone (and subsequently low intratesticular testosterone), and mildly elevated estradiol levels [51]. This hormone profile lends itself to possible manipulation in an effort to increase intratesticular testosterone levels and spermatogenesis. Those with low serum testosterone and elevated estradiol (abnormal ratio of testosterone to estradiol) may have increased levels of aromatase, and therefore off-label use of aromatase inhibitors may increase serum testosterone levels, decrease estradiol, support spermatogenesis and increase intratesticular testosterone [52]. Other strategies for hormone optimization in NOA men include the use of selective estrogen receptor modulators (SERMs) and human chorionic gonadotrophic (hCG). Clomiphene citrate is a SERM that promotes gonadotropin release secondary to competitive binding of the estrogen receptor resulting in increased androgen production, and hCG works to directly stimulate luteinizing hormone (LH) receptors on Leydig cells in the testicle for androgen production [4]. Although normalizing serum testosterone levels (and thereby enhancing intratesticular sperm production) makes sense for men with low testosterone, the proof that such medical intervention helps sperm retrieval rates is limited. Most studies have been non-comparator trials, with the same limitations as noted for varicocele repair prior to TESE. Indeed, our non-randomized results showed that men with low T who were treated had a sperm retrieval rate of 51%, but the men with low T who were not treated had sperm found in 61% of cases (*p* = 0.3) [53].

Sperm processing, as previously described, is also a key aspect for optimization and identification of sperm. Using sharp scissors to finely mince the harvested tissue, followed by repeated aspiration of the tissue homogenate through a 24-gauge angiocath ensures that surgically retrieved tissue is adequately disrupted, and results in up to a 300-fold increase in detectable sperm within minutes of analysis in the operating room [9].

## 7. The Future of Sperm Retrieval in NOA Men

mTESE has been a revolutionary procedure for the treatment of men with NOA. However, there are relatively limited preoperative factors at this time that help to predict sperm retrieval. Therefore, there is ongoing reliance on dedicated surgical examination of testicular tissue in these patients as well as surgeon experience, preoperative optimization (hormone levels and varicocele repair), and teamwork with reliable and experienced laboratory personnel to increase success. Unfortunately, further processing of tissue in the laboratory rarely identifies sperm that was not detected on preliminary examination of a well-digested testicular tissue specimen in the operating room. Moving forward, new technologies for assisted reproduction, including microfluidics, cell sorting, or other micro-identification techniques could permit identification of rare sperm and possibly selection of optimal sperm from a limited pool to improve the likelihood of pregnancy success and live birth rate. Although testicular sperm have 0% normal morphology and are often qualitatively seen to be grossly abnormal, even immotile, from men with NOA, the 43% clinical pregnancy rate obtained with such sperm remains a remarkable reflection of the ability of sperm to contribute to a normal pregnancy with ICSI. Little further improvement in sperm retrieval rates is likely to occur with current surgical techniques, but the learning curve for finding rare sperm is difficult to master, and the care taken to maintain testicular function is critical. Advanced imaging techniques to identify sites of sperm production preoperatively could greatly enhance the application of surgical sperm retrieval. Furthermore, a better understanding of the likely genetic components of NOA may allow non-surgical interventions to enhance sperm production, especially for men with maturation arrest as the presentation of NOA.

## Data Availability

Not applicable.

## References

[1] Agarwal A., Mulgund A., Hamada A., Chyatte M.R. (2015). A unique view on male infertility around the globe. Reprod. Biol. Endocrinol..

[2] Schlegel P.N. (2004). Causes of azoospermia and their management. Reprod. Fertil. Dev..

[3] Kumar R. (2013). Medical management of non-obstructive azoospermia. Clinics.

[4] Esteves S.C. (2015). Clinical management of infertile men with nonobstructive azoospermia. Asian J. Androl..

[5] Fedder J., Crüger D., Oestergaard B., Petersen G.B. (2004). Etiology of azoospermia in 100 consecutive nonvasectomized men. Fertil. Steril..

[6] Matsumiya K., Namiki M., Takahara S., Kondoh N., Takada S., Kiyohara H., Okuyama A. (1994). Clinical study of azoospermia. Int. J. Androl..

[7] Das A., Halpern J.A., Darves-Bornoz A.L., Patel M., Wren J., Keeter M.K., Brannigan R.E. (2020). Sperm retrieval success and testicular histopathology in idiopathic nonobstructive azoospermia. Asian J. Androl..

[8] Krausz C., Riera-Escamilla A., Moreno-Mendoza D., Holleman K., Cioppi F., Algaba F., Pybus M., Friedrich C., Wyrwoll M.J., Casamonti E. (2020). Genetic dissection of spermatogenic arrest through exome analysis: Clinical implications for the management of azoospermic men. Genet. Med..

[9] Schlegel P.N. (2009). Nonobstructive Azoospermia: A Revolutionary Surgical Approach and Results. Semin. Reprod. Med..

[10] Wang J., Sauer M.V. (2006). In vitro fertilization (IVF): A review of 3 decades of clinical innovation and technological advancement. Ther. Clin. Risk Manag..

[11] Temple-Smith P.D., Southwick G.J., Yates C.A., Trounson A.O., De Kretser D.M. (1985). Human pregnancy by in vitro fertilization (IVF) using sperm aspirated from the epididymis. J. Assist. Reprod. Genet..

[12] Palermo G., Joris H., Devroey P., Van Steirteghem A.C. (1992). Pregnancies after intracytoplasmic injection of single spermatozoon into an oocyte. Lancet.

[13] Craft I., Bennett V., Nicholson N. (1993). Fertilising ability of testicular spermatozoa. Lancet.

[14] Enatsu N., Chiba K., Fujisawa M. (2015). The development of surgical sperm extraction and new challenges to improve the outcome. Reprod. Med. Biol..

[15] Devroey P., Liu J., Nagy Z., Goossens A., Tournaye H., Camus M., Van Steirteghem A., Silber S. (1995). Pregnancies after testicular sperm extraction and intracytoplasmic sperm injection in non-obstructive azoospermia. Hum. Reprod..

[16] Schlegel P.N., Palermo G.D., Goldstein M., Menendez S., Zaninovic N., Veeck L.L., Rosenwaks Z. (1997). Testicular sperm ex-traction with intracytoplasmic sperm injection for nonobstructive azoospermia. Urology.

[17] Tash J.A., Schlegel P.N. (2001). Histologic effects of testicular sperm extraction on the testicle in men with nonobstructive azoo-spermia. Urology.

[18] Lewin A., Weiss D.B., Friedler S., Ben-Shachar I., Porat-Katz A., Meirow D., Schenker J.G., Safran A. (1996). Delivery following intracytoplasmic injection of mature sperm cells recovered by testicular fine needle aspiration in a case of hypergonadotropic azoospermia due to maturation arrest. Hum. Reprod..

[19] Schlegel P.N., Li P.S. (1998). Microdissection tese: Sperm retrieval in non-obstructive azoospermia. Hum. Reprod. Update.

[20] Schlegel P.N. (1999). Testicular sperm extraction: Microdissection improves sperm yield with minimal tissue excision. Hum. Reprod..

[21] Ramasamy R., Yagan N., Schlegel P.N. (2005). Structural and functional changes to the testis after conventional versus microdis-section testicular sperm extraction. Urology.

[22] Schlegel P.N., Sigman M., Collura B., De Jonge C.J., Eisenberg M.L., Lamb D.J., Mulhall J.P., Niederberger C., Sandlow J.I., Sokol R.Z. (2021). Diagnosis and treatment of infertility in men: AUA/ASRM guideline part II. Fertil. Steril..

[23] Tournaye H., Verheyen G., Nagy P., Ubaldi F., Goossens A., Silber S., Van Steirteghem A.C., Devroey P. (1997). Are there any predictive factors for successful testicular sperm recovery in azoospermic patients?. Hum. Reprod..

[24] Schlegel P.N., Sigman M., Collura B., De Jonge C.J., Eisenberg M.L., Lamb D.J., Mulhall J.P., Niederberger C., Sandlow J.I., Sokol R.Z. (2021). Diagnosis and Treatment of Infertility in Men: AUA/ASRM Guideline Part I. J. Urol..

[25] Seo J.T., Ko W.-J. (2001). Predictive factors of successful testicular sperm recovery in non-obstructive azoospermia patients. Int. J. Androl..

[26] Raheem A.A., Garaffa G., Rushwan N., De Luca F., Zacharakis E., Raheem T.A., Freeman A., Serhal P., Harper J.C., Ralph D. (2013). Testicular histopathology as a predictor of a positive sperm retrieval in men with non-obstructive azoospermia. BJU Int..

[27] Schoor R.A., Elhanbly S., Niederberger C.S., Ross L.S. (2002). The role of testicular biopsy in the modern management of male in-fertility. J. Urol..

[28] Corona G., Minhas S., Giwercman A., Bettocchi C., Dinkelman-Smit M., Dohle G., Fusco F., Kadioglou A., Kliesch S., Kopa Z. (2019). Sperm recovery and icsi outcomes in men with non-obstructive azoospermia: A systematic review and meta-analysis. Hum. Reprod. Update.

[29] Wang T., Li H., Chen L.-P., Yang J., Li M.-C., Chen R.-B., Lan R.-Z., Wang S.-G., Liu J.-H. (2018). Predictive value of FSH, testicular volume, and histopathological findings for the sperm retrieval rate of microdissection TESE in nonobstructive azoospermia: A meta-analysis. Asian J. Androl..

[30] Bryson C.F., Ramasamy R., Sheehan M., Palermo G.D., Rosenwaks Z., Schlegel P.N. (2014). Severe Testicular Atrophy does not Affect the Success of Microdissection Testicular Sperm Extraction. J. Urol..

[31] Ishikawa T. (2011). Surgical recovery of sperm in non-obstructive azoospermia. Asian J. Androl..

[32] Silber S.J., Van Steirteghem A., Nagy Z., Liu J., Tournaye H., Devroey P. (1996). Normal pregnancies resulting from testicular sperm extraction and intracytoplasmic sperm injection for azoospermia due to maturation arrest. Fertil. Steril..

[33] Ramasamy R., Lin K., Gosden L.V., Rosenwaks Z., Palermo G.D., Schlegel P.N. (2009). High serum fsh levels in men with nonob-structive azoospermia does not affect success of microdissection testicular sperm extraction. Fertil. Steril..

[34] Grunewald S., Glander H.-J., Paasch U., Kratzsch J. (2013). Age-dependent inhibin B concentration in relation to FSH and semen sample qualities: A study in 2448 men. Reproduction.

[35] Chen B., Yang Q., Huang Y.-P., Wang H.-X., Hu K., Wang Y.-X., Huang Y.-R. (2015). Follicle-stimulating hormone as a predictor for sperm retrieval rate in patients with nonobstructive azoospermia: A systematic review and meta-analysis. Asian J. Androl..

[36] Bakircioglu M.E., Erden H.F., Kaplancan T., Ciray N., Bener F., Bahceci M. (2006). Aging may adversely affect testicular sperm recovery in patients with Klinefelter syndrome. Urology.

[37] Bernie A.M., Ramasamy R., Schlegel P.N. (2013). Predictive factors of successful microdissection testicular sperm extraction. Basic Clin. Androl..

[38] Hopps C.V., Mielnik A., Goldstein M., Palermo G.D., Rosenwaks Z., Schlegel P.N. (2003). Detection of sperm in men with Y chromosome microdeletions of the AZFa, AZFb and AZFc regions. Hum. Reprod..

[39] Stahl P.J., Masson P., Mielnik A., Marean M.B., Schlegel P.N., Paduch D.A. (2010). A decade of experience emphasizes that testing for Y microdeletions is essential in American men with azoospermia and severe oligozoospermia. Fertil. Steril..

[40] A Fakhro K., ElBardisi H., Arafa M., Robay A., Rodriguez-Flores J.L., Al-Shakaki A., Syed N., Mezey J.G., Khalil C.A., A Malek J. (2018). Point-of-care whole-exome sequencing of idiopathic male infertility. Genet. Med..

[41] Ramasamy R., Bakircioglu M.E., Cengiz C., Karaca E., Scovell J., Jhangiani S.N., Akdemir Z.C., Bainbridge M., Yu Y., Huff C. (2015). Whole-exome sequencing identifies novel homozygous mutation in npas2 in family with nonobstructive azoo-spermia. Fertil. Steril..

[42] Osaka A., Iwahata T., Kobori Y., Shimomura Y., Yoshikawa N., Onota S., Yamamoto A., Ide H., Sugimoto K., Okada H. (2020). Testicular volume in non-obstructive azoospermia with a history of bilateral cryptorchidism may predict successful sperm re-trieval by testicular sperm extraction. Reprod. Med. Biol..

[43] Ozan T., Karakeci A., Kaplancan T., Pirincci N., Firdolas F., Orhan I. (2019). Are predictive factors in sperm retrieval and preg-nancy rates present in nonobstructive azoospermia patients by microdissection testicular sperm extraction on testicle with a history of orchidopexy operation?. Andrologia.

[44] Glina S., Vieira M. (2013). Prognostic factors for sperm retrieval in non-obstructive azoospermia. Clinics.

[45] Ramasamy R., Padilla W.O., Osterberg E.C., Srivastava A., Reifsnyder J.E., Niederberger C., Schlegel P.N. (2013). A comparison of models for predicting sperm retrieval before microdissection testicular sperm extraction in men with nonobstructive azoo-spermia. J. Urol..

[46] Raman J.D., Schlegel P.N. (2003). Testicular Sperm Extraction with Intracytoplasmic Sperm Injection is Successful for the Treatment of Nonobstructive Azoospermia Associated with Cryptorchidism. J. Urol..

[47] Bernie A.M., Mata D.A., Ramasamy R., Schlegel P.N. (2015). Comparison of microdissection testicular sperm extraction, conventional testicular sperm extraction, and testicular sperm aspiration for nonobstructive azoospermia: A systematic review and meta-analysis. Fertil. Steril..

[48] Colpi G.M., Colpi E.M., Piediferro G., Giacchetta D., Gazzano G., Castiglioni F.M., Magli M.C., Gianaroli L. (2009). Microsurgical TESE versus conventional TESE for ICSI in non-obstructive azoospermia: A randomized controlled study. Reprod. Biomed. Online.

[49] Deruyver Y., Vanderschueren D., Van Der Aa F. (2014). Outcome of microdissection TESE compared with conventional TESE in non-obstructive azoospermia: A systematic review. Andrology.

[50] Ron-El R., Strassburger D., Friedler S., Komarovski D., Bern O., Soffer Y., Raziel A. (1997). Extended sperm preparation: An al-ternative to testicular sperm extraction in non-obstructive azoospermia. Hum. Reprod..

[51] Practice Committee of the American Society for Reproductive Medicine in Collaboration with the Society for Male Reproduction and Urology (2018). Evaluation of the azoospermic male: A committee opinion. Fertil. Steril..

[52] Schlegel P.N. (2012). Aromatase inhibitors for male infertility. Fertil. Steril..

[53] Reifsnyder J.E., Ramasamy R., Husseini J., Schlegel P.N. (2012). Role of Optimizing Testosterone Before Microdissection Testicular Sperm Extraction in Men with Nonobstructive Azoospermia. J. Urol..

